# Inductive bias influences the spatial scale of biological features learned from images

**DOI:** 10.1093/bioadv/vbag238

**Published:** 2026-08-17

**Authors:** Jacob I Evarts, Jason Y Cain, Po-Hao Chiu, Ian Jan, Nancy L Allbritton, Neda Bagheri

**Affiliations:** Department of Biology, University of Washington, Seattle, WA 98195, United States; Department of Chemical Engineering, University of Washington, Seattle, WA 98195, United States; Department of Chemical Engineering, University of Washington, Seattle, WA 98195, United States; Department of Bioengineering, University of Washington, Seattle, WA 98195, United States; Department of Bioengineering, University of Washington, Seattle, WA 98195, United States; Department of Biology, University of Washington, Seattle, WA 98195, United States; Department of Chemical Engineering, University of Washington, Seattle, WA 98195, United States

## Abstract

**Motivation:**

The inductive bias of a deep learning model influences the features it extracts from biological images, making model selection a critical scientific decision. We systematically compare representations learned from scratch without pre-training by convolutional neural networks (CNNs), vision transformers, and Fourier neural operators (FNOs)—an emerging architecture largely unexplored in biological imaging—to determine how their distinct learning mechanisms shape feature learning from spatiotemporal data.

**Results:**

Using a self-supervised representation learning framework on microscopy images of 2D gastruloids, as well as a distinct synthetic tumor image dataset, we show that while CNNs and FNOs achieve comparable accuracy on biological tasks, they learn features at different scales. Visual interpretation methods reveal that CNNs prioritize local, fine-grained details, whereas FNOs capture global, low-frequency structures, in line with overall population morphology. These findings demonstrate that deep learning model architecture is a choice that shapes the biological scale of extracted features, highlighting the need to align a model’s inductive bias with the scientific question.

**Availability and implementation:**

All source code for the representation learning model, the tumor simulation dataset, and the gastruloid microscopy imaging dataset are available at the corresponding DOIs: https://doi.org/10.5281/zenodo.19672838, https://doi.org/10.5281/zenodo.19361370, https://doi.org/10.5281/zenodo.19373112, respectively.

## 1 Introduction

Automated discovery from biological images aims to extract meaningful patterns and hypothesize the mechanisms that underlie emergent behavior. Modern high-throughput live-cell imaging captures time-resolved observations of spatially heterogeneous systems (Weigert *et al.* 2018, Pylvänäinen *et al.* 2023, Qiao *et al.* 2023), producing information-dense datasets that can both enable and complicate this objective (Lähnemann *et al.* 2020, Bagheri *et al.* 2022). While traditional approaches for image analysis have proven effective in measuring attributes like cell size or number, time-resolved live-cell imaging offers new opportunities to uncover nuanced behaviors in the context of their higher resolution dynamics. In order to maximize the knowledge gained from rich spatiotemporal information, automated methods are needed to simplify, interpret, and forecast system-wide dynamics directly from image sequences.

Deep learning approaches, such as convolutional neural networks (CNNs), have demonstrated success in automating tasks like segmentation and feature extraction in microscopy images (Avendi *et al.* 2016, Kan 2017, Sadanandan *et al.* 2017); however, their architecture presents inherent challenges when applied to multiscale analysis. CNNs learn to represent images by detecting spatially localized patterns within sliding windows called kernels. These kernels impose assumptions about how data is organized, a form of *inductive bias*. In CNNs, this bias manifests as a preference for local structure, since each convolutional layer predominantly processes and merges information from small regions in the previous layer. Consequently, early layers of a CNN capture finer spatial details while later layers typically capture semantic features. Although an emphasis on local structure supports the detection of spatial patterns, biological systems exhibit organization across multiple scales, including local interactions such as cell–cell adhesion and mechanical forces (Vopálenská *et al.* 2005, Kannan *et al.* 2025), and global interactions such as nutrient composition or substrate stiffness (Zarkoob *et al.* 2015, D’Souza *et al.* 2021). To capture broader spatial dependencies, vision transformers (ViTs; Dosovitskiy *et al.* 2021) have been adopted widely for biological image analysis (Fu *et al.* 2025, Kenyon-Dean *et al.* 2025). Transformer architectures use self-attention mechanisms to compute pairwise relationships between patches of an image, while CNNs rely on localized receptive fields. While the patches of an image are conceptually similar to stationary kernels in CNNs, the attention mechanism integrates information from regions that are spatially distant without needing to aggregate data over many layers (although network depth improves transformer learning in other ways). As a result, the features being learned by ViT models are relational, representing contextual dependencies between patches. Fourier neural operators (FNOs) offer an alternative approach by utilizing a kernel that operates in the frequency domain. By leveraging the Fast Fourier Transform (FFT), the FNO kernels perform global convolutions in the spectral domain, extracting features based on spectral properties (i.e. Fourier mode content). This mechanism allows FNOs to learn relationships across the entire image by transforming low-frequency modes that capture large-scale structures. The shift from spatial to spectral convolution represents a substantial change in how models process data, transitioning from treating images as discrete grids to modeling them as continuous functions. This architectural design introduces a complementary inductive bias distinct from CNNs and ViTs, favoring smoothness and global correlation. While FNOs have seen growing adoption in the physical sciences—including weather and climate modeling (Kurth *et al.* 2023), chaotic systems (Feng *et al.* 2024), and seismology (Li *et al.* 2023a)—their application to biological imaging remains largely unexplored.

There are compelling theoretical advantages to applying FNOs to biological image analysis. Many fundamental biological processes—including cellular signaling (Weijer 2003), morphogenetic patterning (Maroudas-Sacks and Keren 2021), and tissue growth dynamics (Ruusuvuori *et al.* 2022)—exhibit spatially continuous behaviors that align well with spectral modeling methods, which model patterns in terms of their frequency components. Although the precise mechanistic equations governing these systems are often unknown or intractable, the resulting images can be treated as discrete observations of these underlying continuous processes. FNOs are uniquely suited for this context: rather than relying on explicit physical laws, they constrain the solution space through an inductive bias for global smoothness. Consequently, this bias allows FNOs to effectively model large-scale biological organization even when the underlying mathematical principles are unknown—a common challenge in the field. Based on these properties, we expect that the global, frequency-based operations of FNOs enable them to learn representations that emphasize large-scale, continuous structures such as population morphology, while the local receptive fields of CNNs prioritize high-resolution details.

In this study, we present a systematic comparison of how architectural inductive biases of FNOs, CNNs, ViTs, and baseline multi-layer perceptrons (MLPs) influence the learning of biologically relevant embeddings from time-series imaging data. To conduct this comparison, we employ a self-supervised representation learning framework to distill imaging data into a compact, low-dimensional latent space. Representation learning underpins many modern biological foundation models (Theodoris *et al.* 2023, Chen and Zou 2024) where latent structure supports prediction across diverse tasks, underscoring the importance of understanding how different architectures shape learned representations. We evaluate the quality of representations in three areas: their ability to reconstruct input data, their predictive performance in subsequent evaluations, and their qualitative interpretation. Our evaluation uses a two-dimensional (2D) gastruloid image dataset, as well as a simulated tumor growth dataset, to assess model generalizability across distinct biological systems. These datasets were paired to provide contrasting morphologies: the gastruloids present as relatively large, smooth structures within the imaging frame, whereas the simulated tumors are smaller and exhibit finer, more localized details. Similar approaches have been used to capture underlying biological mechanisms and predict cellular outcomes (Soelistyo *et al.* 2022, Rotem *et al.* 2024), motivating our analysis of how architecture choice influences the biological relevance of learned representations.

Our investigations reveal that FNOs and CNNs learn representations with comparable reconstruction and predictive performance, yet encode distinct aspects of the underlying system. FNOs tend to prioritize smooth, globally coherent structures, whereas CNNs emphasize more localized patterns, often capturing finer details. The ViT underperformed relative to the CNN and FNO architectures based on our evaluated tasks, likely due to the relatively small dataset and lack of pre-training. These results underscore that the architectural bias influences the scale and structure of the extracted representations, and should be treated as an important design factor alongside model capacity and training regimes.

## 2 Methods

### 2.1 Gastruloid culture and microscopy

Gastruloids were grown from RUES2 human pluripotent stem cells (hPSCs; NIHhESC-09–0013) expressing three individual reporters for germ layer fates (SOX2-mCitrine, BRA-mCerulean, and SOX17-tdTomato; Martyn *et al.* 2019). hPSCs were maintained in mTeSR Plus (#100–0276, STEMCELL Technologies) on well-plates coated with 1% Matrigel (#354234, Corning Inc.) in Advanced DMEM/F12 (#12634–010, Gibco). Cells were routinely passaged using Accutase (#07920, STEMCELL Technologies) with mTeSR Plus supplemented with 10 μM Rho-associated kinase inhibitor Y-27632 (#B1293, APExBIO Technology LLC). To induce aneuploidy, cells were treated with 0.5 μM reversine (#10004412, Cayman Chemicals Co.) for 48 hours with a media change at 24 hours. 2D gastruloids were cultured by confining hPSCs on micropatterned arrays created by exposing PLL-g-PEG-coated arrays to deep UV light through a custom photomask (Jan *et al.* 2025). hPSCs were replated on the arrays in mTeSR Plus containing 50 ng/mL bone morphogenetic protein 4 (BMP4; #314-BP-010, R&D Systems) and incubated for 48 hours.

**Figure 1:**
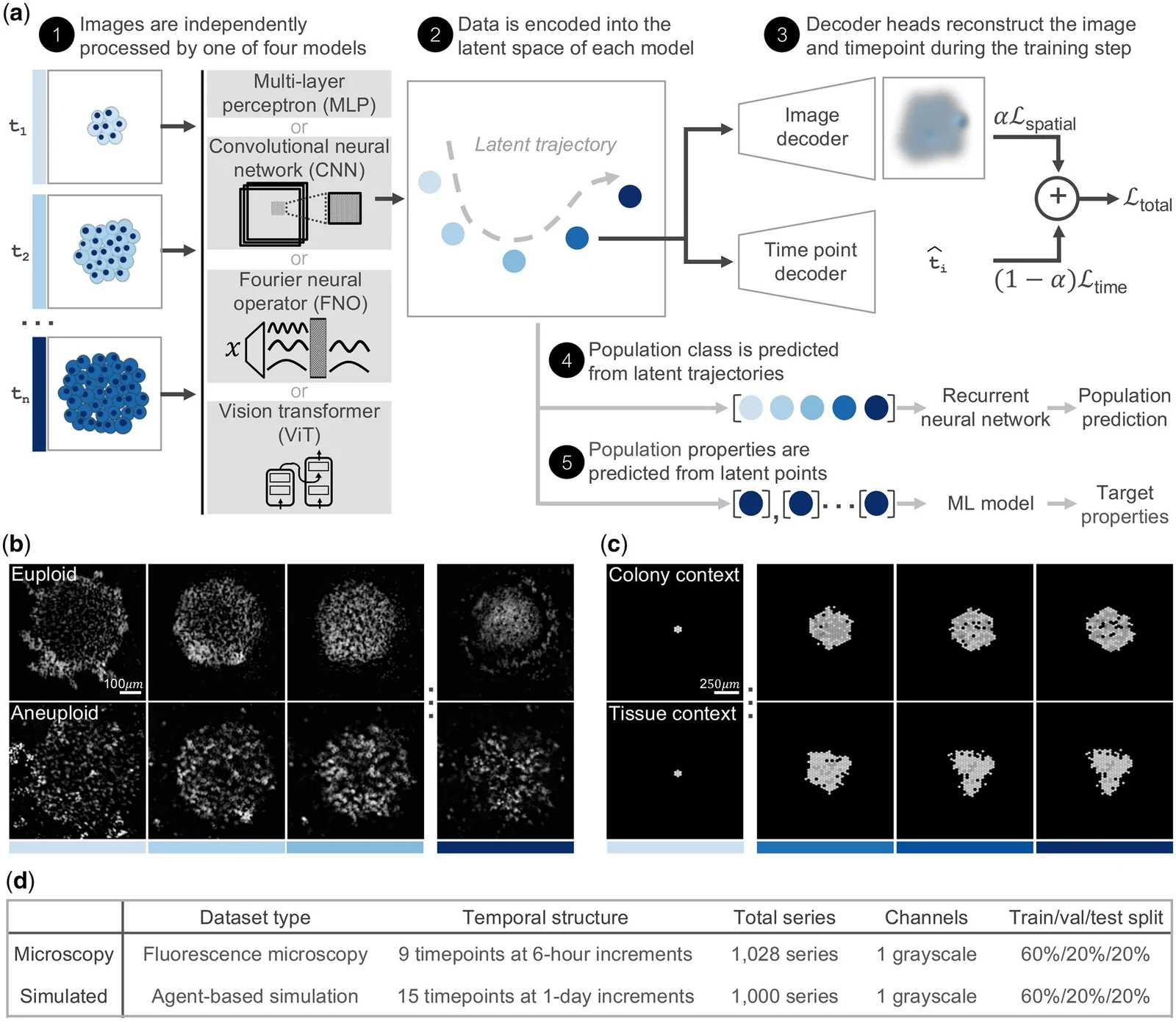
A spatiotemporal autoencoder framework for learning representations from biological image series. (a) Overview of the autoencoder architecture designed to learn representations of biological images. (1) Individual images from a time-series ($t_{0},\ldots ,t_{n}$) are passed to each of four parallel encoders: an MLP, a CNN, an FNO, or a ViT. (2) The encoder compresses each image to a low-dimensional latent vector, forming a latent trajectory. (3) For training, the model uses these latent vectors for two reconstruction tasks: an image decoder reconstructs the input image and a time point decoder predicts the relative position the image was taken in its sequence. The total loss ($L_{\mathrm{total}}$) is a weighted sum of the spatial reconstruction loss ($L_{\mathrm{spatial}}$) and the temporal prediction loss ($L_{\mathrm{time}}$), given by $L_{\mathrm{total}}$ = α($L_{\mathrm{spatial}}$) + (1−α)($L_{\mathrm{time}}$). (4) The utility of the learned trajectories is evaluated by training a sequence model that classifies the population. (5) The representations are further tested by predicting continuous morphological metrics. (b) Representative microscopy images from the gastruloid dataset. The top row shows a developing euploid gastruloid and the bottom row shows an aneuploid gastruloid over time. (c) Representative images from a simulated dataset of tumor growth. The top row (colony context) shows a tumor growing in isolation, while the bottom row (tissue context) shows a tumor growing with a healthy cell background. (d) Table providing details on the datasets used in this study.

Time-lapse microscopy was performed using a custom system built around an inverted MVX10 MacroView fluorescence microscope (Evident Scientific) enclosed within an incubation chamber (Jan *et al.* 2025). Illumination was provided by a Lumen 200 mercury lamphouse (Prior Scientific Inc.) through an 8907 ET—ECFP/EYFP/DsRed filter set (Chroma Technology Corp). Images were acquired at 4× magnification (1.7 μM/pixel). The images were taken every 6 hours for 2 days (48 hours), resulting in nine frames per gastruloid. A total of 1058 gastruloids were grown, evenly split between euploid and aneuploid conditions, resulting in 9252 images (Fig. 1d).

Raw microscopy images were preprocessed before being used to train the model. To increase contrast and reduce background lighting effects, we applied Gaussian blur (σ= 50) and background removal before normalization. The dataset was divided into training (60%), validation (20%), and test (20%) sets, with all images from a single gastruloid assigned to the same set to prevent data leakage.

### 2.2 Generation of *in silico* tumor growth dataset

Synthetic data of tumor growth dynamics were generated using ARCADE, an agent-based model (ABM) of the tumor microenvironment that simulates heterogeneous vasculature, nutrient diffusion, and distinct cell-type behaviors (Yu and Bagheri 2020). The complete data set comprises 1000 full simulations: 500 *in vitro*-like colony simulations and 500 *in vivo*-like tissue simulations with a healthy cell background. The images were rendered from the simulation at day intervals, resulting in 15 frames capturing the population dynamics over 15 days. The simulations used here closely follow those described in (Yu and Bagheri 2021). This regime resulted in 15 000 images, where each image is a 128×128 pixel grayscale representation of the simulation, with pixel intensity representing the number of cancer cells at given grid locations. The dataset was partitioned into training (60%), validation (20%), and test (20%) sets, ensuring images from the same simulation remained within one set to prevent data leakage.

### 2.3 Autoencoder architecture and training

To learn representations that capture both spatial and temporal structure from image sequences, we designed an autoencoder framework with a single encoder and two decoder heads (Fig. 1a). The encoder maps an input image to a latent vector, which is used by a spatial decoder to reconstruct the original image and by a temporal decoder to predict the relative time point of the image. The two decoder losses are combined [see (1)] to form a shared objective that encourages the latent space to jointly support image reconstruction and temporal ordering. By processing each image independently, this design avoids more complex sequential architectures and allows for the separate tuning of spatial and temporal structure via the weight α. For consistency across the baseline comparisons, α was fixed at 0.9 for all architectures evaluated. The encoders were constructed using one of four architectures: (i) a MLP where input images were flattened and processed by fully connected layers; (ii) a CNN with a series of convolutional blocks and max-pooling layers; (iii) a ViT comprising a patch embedding layer, corresponding positional encodings, and a transformer block utilizing multi-head self-attention; and (iv) a FNO that performed global convolution in the frequency domain. Each model was trained from scratch with randomly initialized weights and without pre-training or transfer learning. Four independent replicates of each architecture were trained, each using a different random weight initialization and train/validation/test data split. All model variants shared the same decoder architectures: an image decoder composed of linear layers with upsampling, and a shallow MLP for the time point decoding. Model complexity was kept comparable across architectures by using relatively shallow designs (e.g. four FNO layers, four convolutional layers, one transformer block) and matching trainable parameter count where feasible (Fig. 1, available as supplementary data at *Bioinformatics Advances* online), a common proxy for model complexity (Myung 2000, Spiegelhalter *et al.* 2002).

All models were trained by minimizing a combined loss function:


(1) $$L_{\text{total}}=\alpha L_{\text{spatial}}+(1-\alpha )L_{\text{time}}$$

where $L_{\text{spatial}}$ is the pixel-wise mean squared error (MSE) between the original and reconstructed images, $L_{\text{time}}$ is the cross-entropy loss for discrete time point prediction, and α is a hyperparameter balancing the two objectives. An exhaustive grid search was conducted over the optimizer hyperparameters, the latent space dimensionality *d*, and architecture details to minimize the combined spatiotemporal validation loss (Table 1). A latent dimension of d=16 was selected as it offered a good trade-off between validation performance and parsimony, and constrained the latent feature space to reduce redundancy. Models were optimized using the Adam optimizer (Kingma and Ba 2017). All selected hyperparameter values for each model across both datasets are detailed in Table 1, available as supplementary data at *Bioinformatics Advances* online. All training and experiments were conducted with PyTorch 2.5.1 (Paszke *et al.* 2019) on a MacBook Pro with an M1 Pro processor utilizing Metal Performance Shaders (MPS) for GPU acceleration.

**Table 1:** Hyperparameters searched during autoencoder optimization.

		Bounds		
	Hyperparameter	Lower	Upper	Type
Model	image loss weight α	0.0	1.0	Linear
	latent dim *d*	2	32	Integer
Adam	learning_rate	0.0001	0.01	Log
	beta_1	0.8	0.99	Linear
	beta_2	0.9	0.999	Linear
SGD	learning_rate	0.0001	0.01	Log
	momentum	0.0	0.9	Linear
	nesterov	False	True	Categorical

The optimizer type (Adam or SGD) was itself included in the search.

### 2.4 Representation evaluation

We evaluated the utility of the learned representations through two tasks targeting different biological predictions: binary classification of latent trajectories and the regression of biological and technical variables. Although classification tasks are commonly used to evaluate latent space utility (Wang and Wang 2019, Mascolini *et al.* 2022, Geleta *et al.* 2023), including regression tasks helps ensure the results are not driven by biases in the downstream models.

For each dataset, the latent vectors generated by the trained encoders were first transformed using principal component analysis (PCA) to obtain an orthogonal basis ordered by variance explained. To prevent data leakage during subsequent evaluation tasks, the PCA model was fit exclusively on the latent vectors generated from the training set images and then applied to transform the validation and test sets. While variance explained does not guarantee biological relevance, an orthogonal basis facilitates the interpretation and perturbation of uncorrelated dimensions. The resulting principal components (PCs), which formed temporally ordered trajectories for each gastruloid or tumor simulation, were then used as input to a sequence classifier. We trained a long short-term memory (LSTM) network to classify the trajectories and evaluated classifications using macro-F1 score and balanced accuracy. For the gastruloid dataset, the task was to classify each trajectory as euploid or aneuploid. For the synthetic tumor dataset, the task was to classify each trajectory as grown with or without a healthy cell background. A hyperparameter search was performed to determine the optimal LSTM architecture for the sequential models (Table 2).

**Table 2:** Hyperparameter search space for the sequence models.

Hyperparameter	Values
Model type	LSTM
hidden_size	32, 64, 128
num_layers	1, 2, 3

To evaluate static feature representations, regressors were trained on the latent PCs from each dataset’s final time point to predict morphological metrics and dynamics. For the gastruloid dataset, the mean intensity, area, circularity, and skew of each gastruloid were predicted. These values were computed by segmenting each gastruloid endpoint image using Fiji (Schindelin *et al.* 2012). For the synthetic tumor dataset, the growth rate, diameter, cell counts, average cell volume, cell activity, and symmetry were predicted. All of these values are tracked over time for each ARCADE simulation (Yu and Bagheri 2020). A grid search was conducted over four types of models, linear regression, elastic net, random forest, and an MLP, to predict the target variables (Table 3).

**Table 3:** Hyperparameter search space for the static regression models.

Model	Hyperparameter	Values
Linear regr.	fit_intercept	True, False
Elastic net	α	0.01, 0.1, 1.0
	l1_ratio	0.1, 0.5, 0.7, 0.9
Random forest	n_estimators	10, 25, 50
	max_depth	10, 20
	min_samples_split	2, 5
	min_samples_leaf	2, 4
MLP	hidden_layer_sizes	50, 100
	learning_rate	constant, adaptive
	α	1e-4, 1e-2
	activation	ReLU, Tanh

### 2.5 Saliency-based feature visualization

To identify which regions of the input image contributed most heavily to the learned representations, we generated saliency maps using Gradient-weighted Class Activation Mapping (Grad-CAM; Selvaraju *et al.* 2020). Adapting this technique from its original classification use case, we targeted the gradient calculations toward the spatial reconstruction loss (pixel-wise MSE between the input and reconstructed images). Gradients of this loss with respect to the activations of the final spatial layer in the encoder (i.e. the final layer before pooling and flattening) were aggregated to produce heatmaps of regions with the strongest effect on reconstruction.

### 2.6 Counterfactual latent space analysis

To compare representations learned by each architecture, we employed a perturbation-based analysis to visualize the spatial information encoded within the most predictive dimensions of the latent space. First, using the PCA transformation fit on the training set, we transformed the full set of latent representations and used the resulting PCs as features to train an LSTM network for the subsequent evaluation task of population classification. To identify the most biologically relevant PCs, we performed a permutation feature importance test where each PC’s values were randomly shuffled, and the resulting drop in classification performance was measured. Finally, for each of the six top-ranked PCs according to the permutation test, we perturbed a sample latent vector by ±3 standard deviations (σ) along that PC’s axis while holding all other PC values constant. This perturbed vector was then transformed back from PC space to the original latent space coordinates and passed through the corresponding trained image decoder. The resulting reconstructed image visually represents the feature encoded by that specific PC.

## 3 Results

We compare the performance of MLP, CNN, ViT, and FNO-based autoencoders to assess how inductive bias shapes the features learned from spatiotemporal imaging data. To isolate the effects of architectural differences, all networks were restricted to simple designs with limited capacity, ranging from 2.74 to 2.79 million trainable parameters (Fig. 1, available as supplementary data at *Bioinformatics Advances* online). Each architecture is implemented within a self-supervised representation learning autoencoder designed to capture both spatial and temporal information (Fig. 1a). In this framework, an encoder network compresses an input image into a low-dimensional latent vector that is then passed through two separate decoder heads. One decoder reconstructs the original input image, with its performance measured by a spatial loss function (i.e. reconstruction error), while another decoder predicts the image’s timestamp, evaluated by a temporal loss function (i.e. classification error). By training the model to minimize a weighted sum of the spatial and temporal losses, we incentivize the latent space to jointly learn the spatial and temporal structure of the underlying biological process.

We evaluate this framework on two distinct case studies. The first is a microscopy dataset of developing human 2D gastruloids, an *in vitro* system that mimics aspects of early embryogenesis, which allows us to assess how well the models distinguish between euploid (healthy) and aneuploid (chromosomally abnormal) populations based on the live reporter signal for the cell lineage marker, SOX2 (Fig. 1b). The second case involves a simulated dataset of tumor growth generated using a multiscale, multiclass ABM (Yu and Bagheri 2020). This dataset serves as a controlled environment to test the models’ ability to differentiate growth dynamics in the presence or absence of competing healthy tissue (Fig. 1c). These datasets enable model architecture evaluation at differing structural scales, as the gastruloids appear as large, smooth colonies, whereas the simulated tumors are smaller in frame and have fine, sometimes jagged morphologies. For both systems, we evaluate the architectures according to three criteria: (i) the accuracy of the spatiotemporal reconstructions, (ii) the latent representation performance in classification and regression tasks, and (iii) the qualitative visual characteristics of the latent features.

### 3.1 All architectures achieve comparable spatiotemporal reconstruction

**Figure 2:**
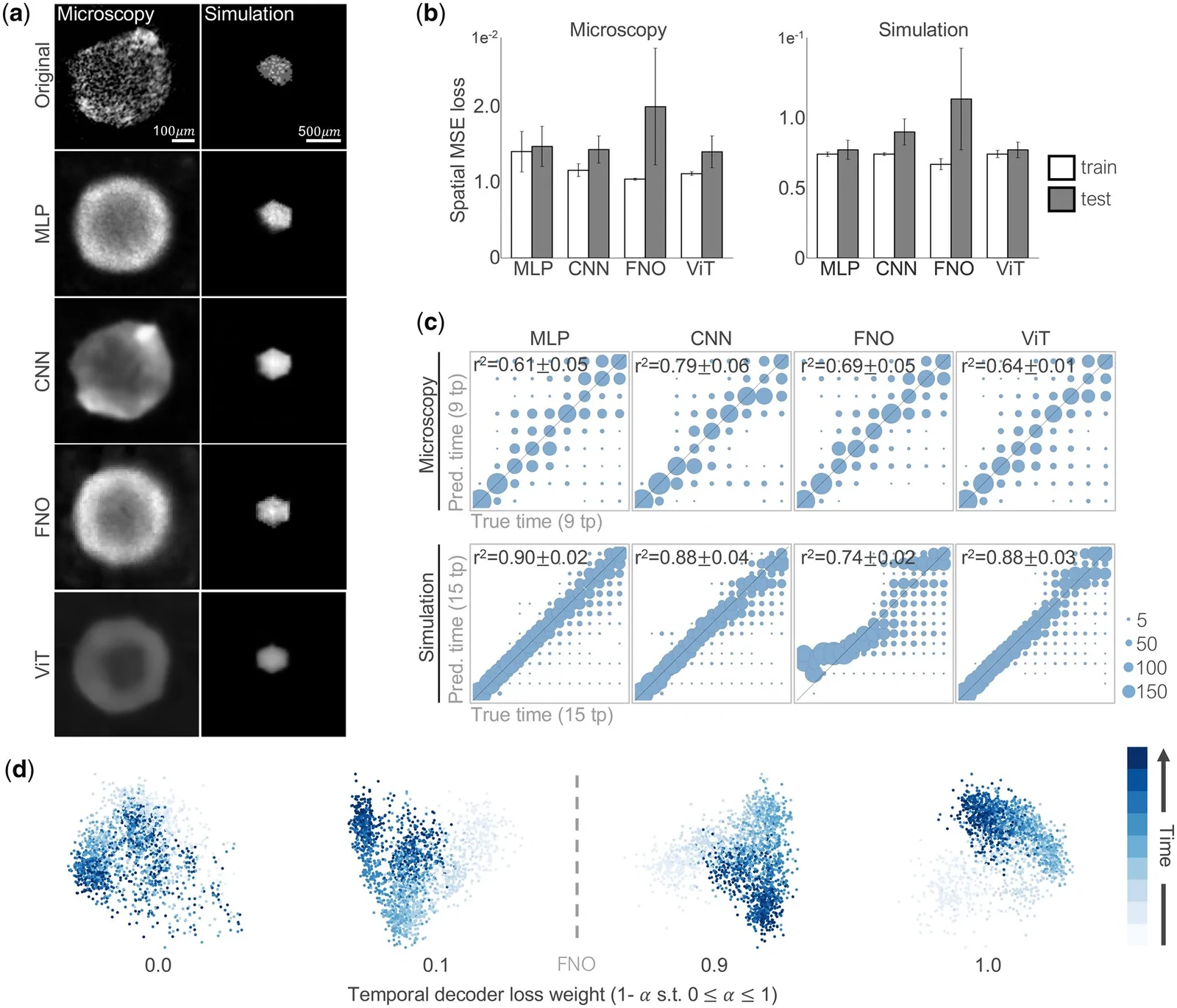
Autoencoder architectures reconstruct microscopy and simulated cell colonies. (a) Representative original images (top row) and their reconstructions from the MLP, CNN, FNO, and ViT models on the gastruloid microscopy and simulated tumor test sets. Models used a 16D latent space. (b) Spatial MSE on the train (white) and test (gray) sets for each encoder type show comparable performance between models, although the FNO produced marginally higher test loss on both datasets. Bars indicate the mean over independent replicates (n=4), with error bars showing the standard deviation. The *y*-axis values for the microscopy and simulated plots are scaled by $10^{-2}$ and $10^{-1}$, respectively. (c) Parity plots of predicted versus true time point on the test sets for microscopy (top) and simulated (bottom) data show comparable performance for all models across both datasets. The coefficient of determination ($r^{2}$) is the mean ± the standard deviation across replicates (n=4). Point size is proportional to the density of data points. (d) PCA projections of the FNO-generated latent space of the microscopy dataset, showing the effect of the temporal loss weight (1−α). Points are colored by their true time point. Minimal temporal information is needed for a chronological ordering of the latent space to emerge. Unless otherwise noted, a weight of 1−α = 0.1 is used in all other experiments.

To assess each architecture’s ability to encode and reconstruct spatial and temporal information from the input images, we record loss data and prediction results from each of the decoder heads (Fig. 2). On the gastruloid dataset, all four architectures achieve comparable spatial reconstructions as evident from the low pixel-wise MSE and agreement in the overall size and shape of the cell population in the reconstructed images (Fig. 2a and b). Qualitatively, the CNN-based model captures fine-grained details, such as distinct cell clusters on the population periphery, more effectively than the other models (Fig. 2a). In addition to spatial reconstruction, all models show a comparable capacity to predict the time point at which each image was taken, demonstrating that the learned latent representations encode temporal progression (Fig. 2c). Furthermore, only a small weight on the temporal decoder head (1−α=0.1) is required to produce smooth latent trajectories and accurate time point predictions (Fig. 2c and d), indicating that only a relatively small amount of the total capacity of the latent dimensions is needed to capture the temporal information. Together, this fidelity demonstrates that all four models can effectively capture spatiotemporal characteristics for continuous, relatively smooth cell populations. The models exhibit varying training times, with the FNO being the most computationally expensive to train, likely due to the lack of hardware-level optimization that the other models benefit from (Table 4).

**Table 4:** Average training runtimes and standard deviations per epoch for each model architecture across both datasets.

Model	Simulation	Microscopy
MLP	22.3±2.2s	14.3±0.8s
CNN	42.0±1.7s	24.1±0.3s
FNO	100.2±2.5s	64.4±2.8s
ViT	25.0±1.2s	12.3±0.5s

Runtime evaluations were performed on a MacBook Pro with an M1 Pro processor utilizing MPS for GPU acceleration.

On the simulated dataset, reconstruction performance from the latent space is similar. The ViT and MLP both achieve low reconstruction error, although the ViT appears to capture cell density (depicted by intensity) better, while the MLP’s performance is possibly due to its ability to characterize the irregular, sometimes jagged colony boundaries characteristic of the ABM’s hexagonal grid environment (Fig. 2a and b). The FNO has the highest test loss on this task, likely because its bias for smooth structures struggles with the irregular colony boundaries that the MLP could model effectively. Despite differences in spatial reconstruction quality, all architectures demonstrate a high degree of accuracy in predicting the time points for the simulated data—a simpler problem than the microscopy data due to the largely monotonic increase in colony size over time (Fig. 2c). While all architectures demonstrate the ability to reconstruct time points, the FNO model had lower fidelity on the simulation data, indicating an inherent difficulty in tracking fine-grained details over time. These findings highlight the sensitivity of each model’s performance to the specific structural characteristics of the input data, even within the same general domain.

### 3.2 Learned representations enable population classification

**Figure 3:**
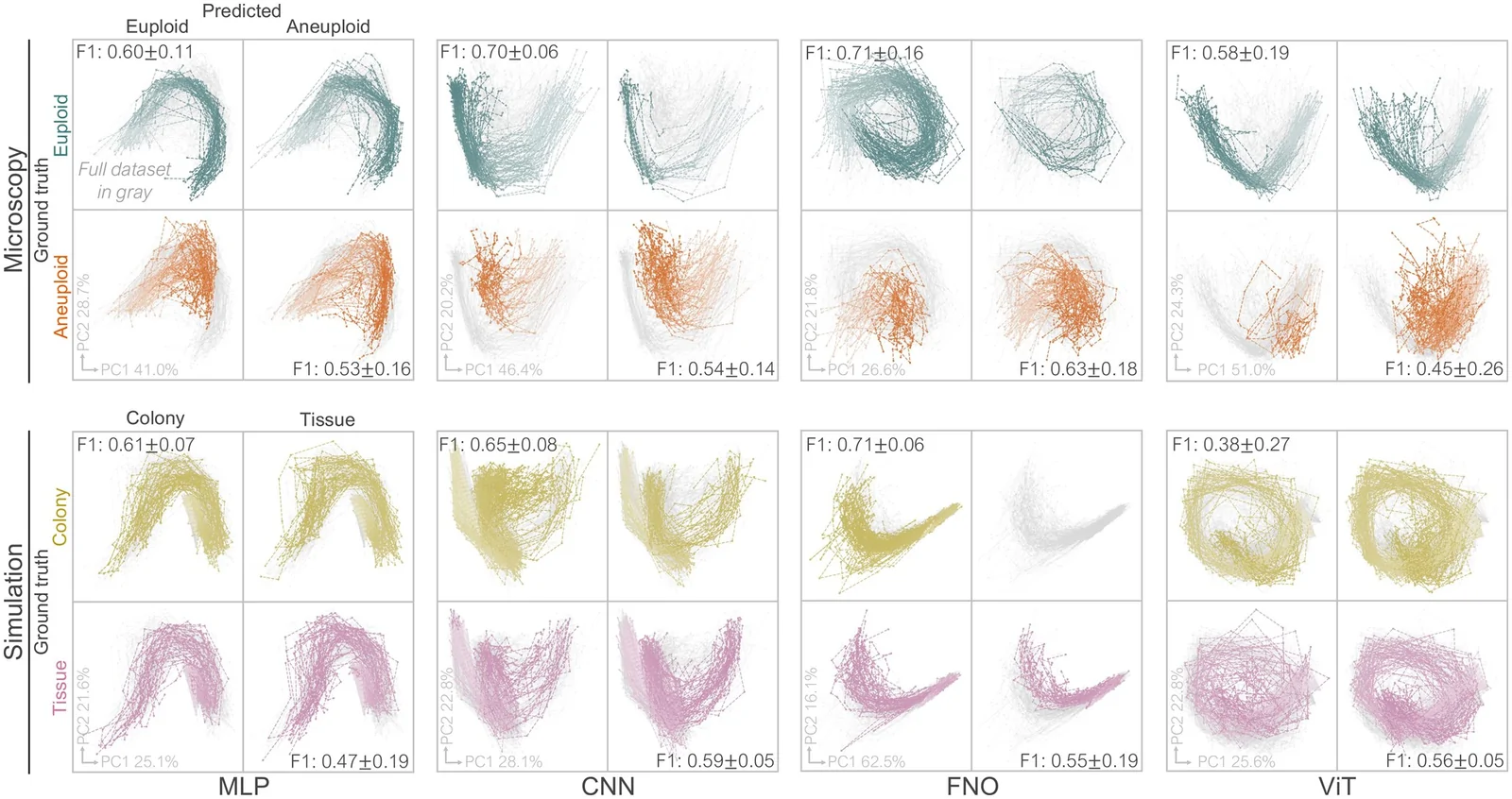
FNO and CNN representations are more predictive for population classification. Performance on classification tasks using the latent trajectories generated by each encoder (MLP, CNN, FNO, ViT). LSTM classifiers were trained to predict population-level labels for the microscopy dataset (top, euploid versus aneuploid) and the simulated dataset (bottom, colony versus tissue). The grid acts as a visual confusion matrix: rows correspond to the ground truth label and columns correspond to the predicted label. Each subplot shows the latent trajectories from the test set, colored by their true class. To illustrate the relative position of each class, points from the contrasting class are shown in light gray in the background of each subplot. The average per-class F1-score is reported along with the standard deviation across replicates (n=4).

A primary goal of representation learning is to extract features that are useful for biological tasks. We use PCs of the latent space of each trained autoencoder to generate temporal trajectories, which then serve as input to LSTM networks (Hochreiter and Schmidhuber 1997) trained to perform population-level classification. To visualize the basis for each classifier’s performance, we plotted the latent trajectories in a confusion matrix-style layout, with correctly classified examples on the diagonal (Fig. 3). Quantitative counts of class predictions for each model are provided in Fig. 2, available as supplementary data at *Bioinformatics Advances* online.

Notably, spatial reconstruction fidelity was not a reliable indicator of downstream predictive performance. Both the MLP and ViT models achieved low spatial reconstruction losses, yet their learned representations struggle to differentiate population classes, with the MLP and ViT representations showing reduced performance on both datasets as measured by macro-F1 score and balanced accuracy (Table 5). Conversely, the CNN and FNO representations had better predictive power on both datasets despite the FNO’s marginally higher spatial reconstruction loss. We examined the relationship between reconstruction loss and classification performance across all of the model architectures and found that neither the spatial nor temporal reconstruction loss were strongly correlated with the classification F1-score (Fig. 3, available as supplementary data at *Bioinformatics Advances* online). This result suggests that the features most important for accurate image reconstruction are not necessarily the same as those that are most biologically informative in distinguishing population-level phenotypes. It is important to note that the training, validation, and test trajectories in the synthetic tumor dataset are produced by the same underlying simulation framework, meaning that learned representations may encode some simulator-specific structure rather than more general biological features.

**Table 5:** Trajectory classification performance across encoder architectures.

	Microscopy		Simulation	
Encoder	Macro-F1	Bal. acc.	Macro-F1	Bal. acc.
MLP	0.57±0.11	0.59±0.10	0.54±0.09	0.56±0.08
CNN	0.62±0.08	0.64±0.07	0.62±0.06	0.63±0.06
FNO	0.67±0.15	0.69±0.13	0.63±0.10	0.65±0.08
ViT	0.51±0.21	0.53±0.20	0.47±0.13	0.51±0.11

Values are mean ± standard deviation over training seeds (n=4).

Despite their comparable classification performance, the latent space organizations of the CNN and FNO models were visually distinct. For the gastruloid data, the FNO-based trajectories loop, whereas the other models have horseshoe-shaped trajectories (Fig. 3, top row). The representations of the simulated tumor dataset also show distinct latent organizations between models, with the exception of the MLP, which appears to learn similarly shaped latent spaces between the two datasets. This contrast suggests that, while each architecture captures relevant biological information, they do so by learning distinct types of features, prompting a deeper analysis into each model’s representation space.

### 3.3 Predicting emergent targets reveals task-specific architectural trade-offs

**Figure 4:**
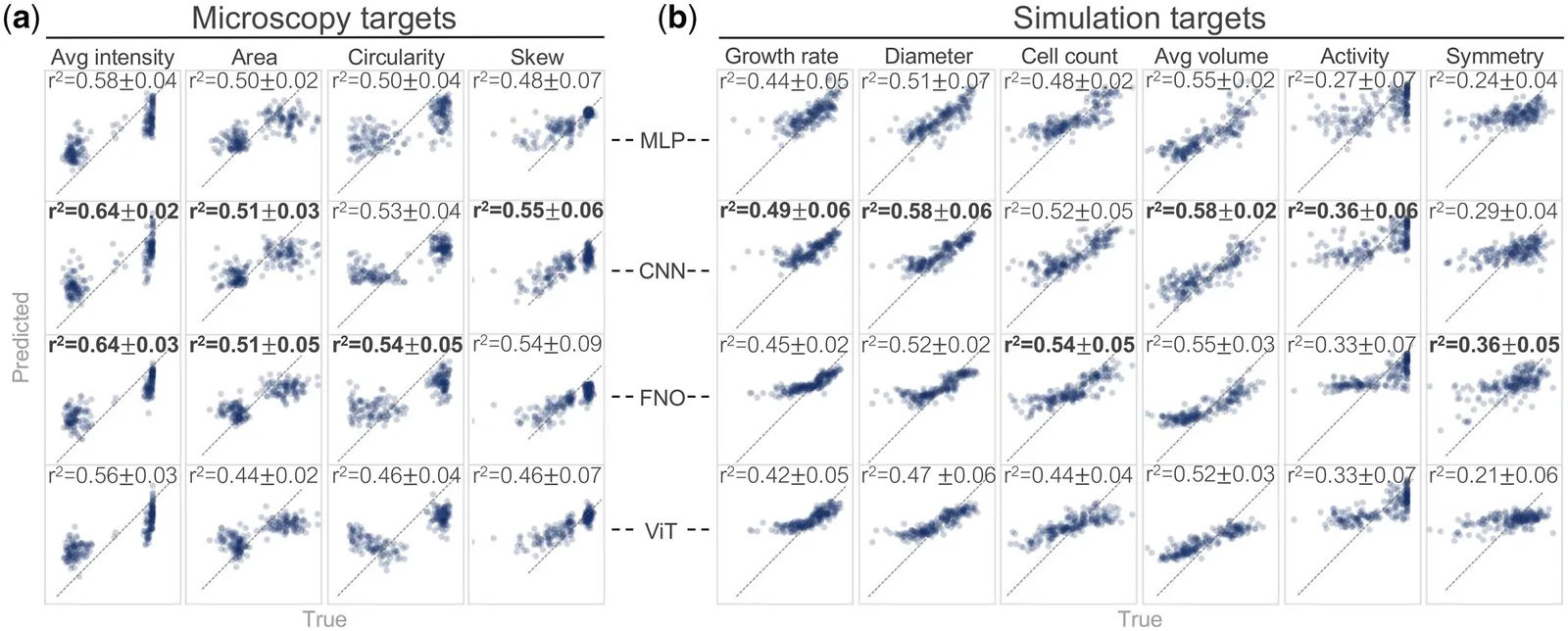
Predicting morphological metrics reveals scale-dependent architectural trade-offs. (a) Parity plots comparing true versus predicted values for continuous morphological metrics in the microscopy dataset. Regression models were trained on the PCs of the final time point latent embeddings. The CNN and FNO-based representations achieved the highest mean $r^{2}$ across all metrics, notably in predicting average intensity. Reported $r^{2}$ values are the mean ± standard deviation across four independent replicates. Bold text indicates the highest $r^{2}$ value for each respective metric. (b) Parity plots for the simulated tumor dataset metrics. Predictive performance differs across metrics, with the CNN representations achieving the highest mean $r^{2}$ across several metrics including tumor diameter and the FNO representations performing relatively better on tumor symmetry. Reported $r^{2}$ values are the mean ± standard deviation across four independent replicates.

To further interrogate the quality of the representations learned by each architecture, we trained regression models on the PCs of the final time point latent embeddings to predict continuous, population-level morphological variables. On the gastruloid dataset, we predicted the average intensity, area, circularity, and skew of each gastruloid. Although predictive accuracy was largely comparable across the models, the FNO and CNN-based representations achieved the highest coefficient of determination ($r^{2}$) values for every target, with skew and average intensity seeing the largest performance differentials between models (Fig. 4a). For the simulated tumor dataset, we predicted the population growth rate, diameter, total cell count, average cell volume, tumor activity, and symmetry (Yu and Bagheri 2020). While baseline performance across these targets remained comparable among the models, the CNN-based representations consistently had the highest performance, with all of the models struggling to predict tumor activity, which is not inherently spatial (Fig. 4b). It is worth noting that even some spatial emergent targets, such as the symmetry of the simulated tumors, were difficult for all the models to predict, giving some insight into the limits of what type of information was learned from the provided loss function.

### 3.4 Architectural inductive bias predisposes models to distinct feature scales

**Figure 5:**
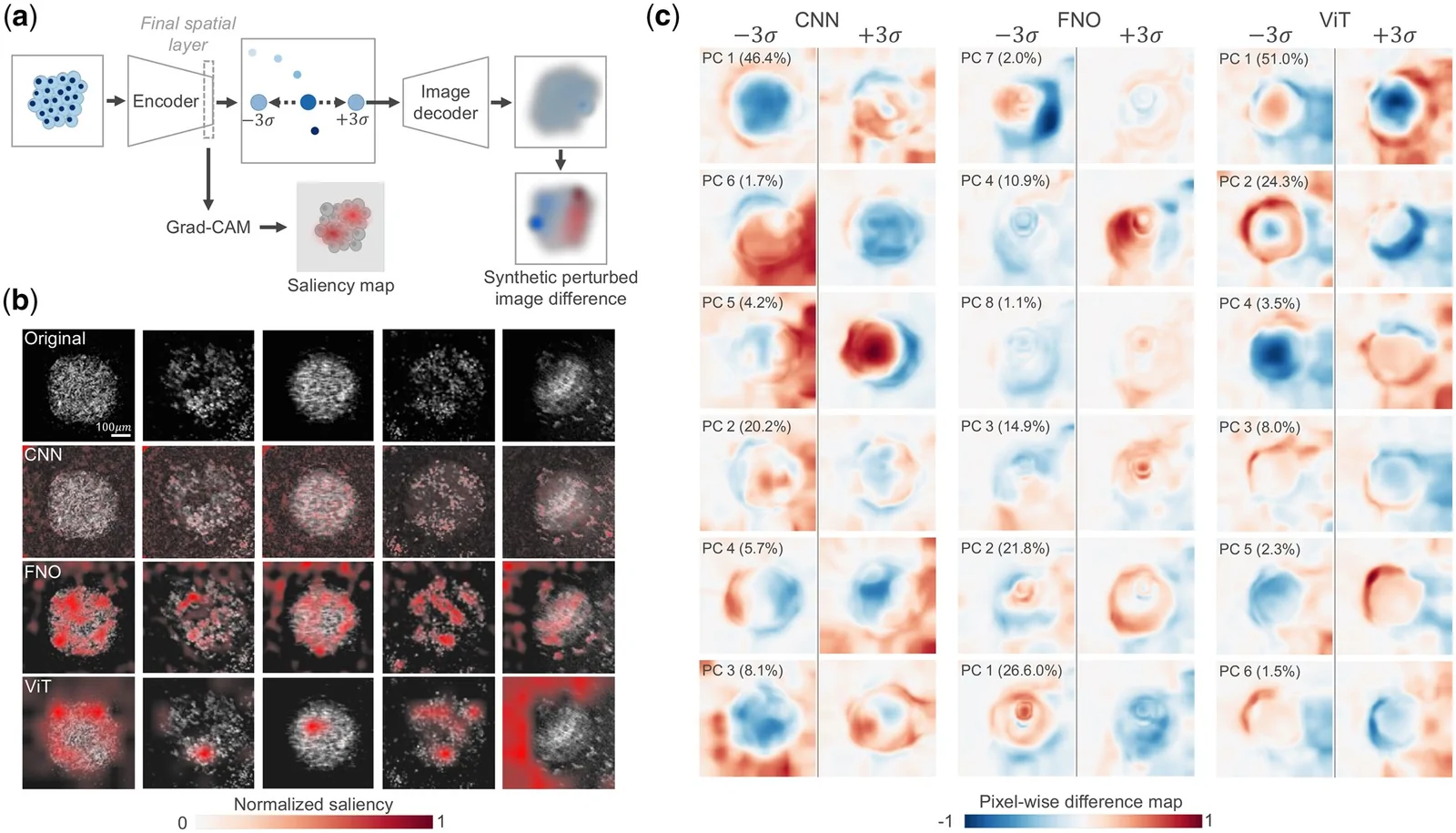
Learned feature scale and saliency differ substantially between encoder architectures. (a) Overview illustration of the visual interpretation methods. Grad-CAM is used to generate saliency maps that highlight important input pixel regions from the final convolution/neural operator layer of the encoder. Latent space vectors are perturbed along their PC axes (±3σ) and then passed through the image decoder to generate synthetic images. The difference between the perturbed and original decoded images reveals which feature a particular axis captures. (b) Saliency maps for the CNN, FNO, and ViT models on five representative microscopy images. Normalized saliency, represented by the heatmap in bottom three rows, is overlaid on the original image (top row). The CNN is prone to focus on numerous small, isolated regions. The FNO is prone to consider broader swaths of the images, including areas outside the gastruloid population. The ViT has less consistent saliency. Scale bar, 100 μM. (c) PCs are ranked by their predictive power for population classification using a permutation-based feature importance measure. For all models, we select the six most predictive PCs. Each of these PCs is individually perturbed by ±3 σ and reconstructed through the image decoder. The resulting effect is visualized as a pixel-wise difference map between the original and perturbed reconstructions. The percentage of total variance explained by each PC is noted in parentheses.

To understand how the FNO and CNN achieve similar classification success from dissimilar representations, we perform both saliency-based and counterfactual-based analysis to visually interpret the encoded features (Fig. 5a). Saliency maps, generated similarly to Grad-CAM (Selvaraju *et al.* 2020), suggest qualitative differences in the spatial distribution of model sensitivity. The CNN exhibited speckled saliency, characterized by numerous small, spatially distributed activations across the image, consistent with local sliding-window feature extraction (Fig. 5b). In contrast, the FNO generally produced broader, more spatially contiguous saliency spanning larger portions of the cell population. The FNO also exhibits a recurring focus on the upper-left corner of some images. Because similar, though less frequent, patterns were observed in other architectures, we cannot rule out that this reflects a technical artifact rather than biologically meaningful structure. Finally, the ViT model has a less consistent pattern, with saliency ranging from focused on cell groupings to less biologically relevant features such as the negative space around the population.

We further examined the latent spaces using a counterfactual analysis to visualize the learned features more globally, similar to methods described previously (Soelistyo *et al.* 2022, Rotem *et al.* 2024). Using a permutation-based feature importance analysis, we selected the most informative PCs from each latent space for population classification. Each of these selected PCs was perturbed ±3 σ before being passed back through the decoder to generate a synthetic image. This analysis revealed some nuanced distinctions in the structure of the features learned by each architecture. Perturbing the CNN’s top predictive PCs tended to reveal complex, intermixing patterns, although some larger structures are also present (Fig. 5c). The FNO’s top predictive PCs tended to be radially organized around a central structure, likely related to the spectral nature of its learning mechanism. Interestingly, more so than the other two models, the ViT’s top predictive PCs more clearly encoded the population shape, with similar structures recurring across multiple PCs (i.e. PC3, PC5, and PC6). This interpretation suggests that the ViT captures fewer diverse modes of variation in the gastruloids than the CNN and FNO, which could explain its weaker performance in subsequent evaluations. We observed similar qualitative patterns across a small sweep of hyperparameter settings for each model, although they were generally less distinct (Fig. 4, available as supplementary data at *Bioinformatics Advances* online).

Taken together, these results provide visual evidence that each architecture encodes different information, likely in part a consequence of their inductive kernel biases. These qualitative analyses suggest that the global, Fourier-based convolutions of the FNO place greater emphasis on radial population structure, whereas the local kernels of the CNN place greater emphasis on more granular details.

## 4. Discussion

### 4.1 Aligning model architecture with biological scales of interest

In this study, we investigate how the inductive biases of different neural network architectures shape the latent representations they learn from *in vitro* and synthetic biological imaging data. Our findings demonstrate that while CNNs and FNOs can achieve comparable predictive performance on population-level classification and regression tasks, they do so by encoding different types of information at different spatial scales. This distinction highlights that the choice of model architecture is not merely an operational detail but a critical decision that shapes the biological insights extracted. Although many model architectures can be tuned to capture a range of structures through hyperparameters such as kernel size, patch size, or Fourier modes, we observed that each architecture’s inductive bias predisposes it to learning certain spatial scales. FNOs, along with other models that exhibit bias toward smooth, large-scale structure, are well suited for studying global phenomena such as collective cell migration, tissue patterning, and colony morphology. The FNO’s ability to filter high-frequency noise and artifacts, to which other models might overfit, may also produce a denoised representation of the overarching biological structure. In contrast, for tasks requiring the detection of localized details or high-frequency patterns, such as cell or subcellular organelle segmentation, a CNN’s established proficiency in learning local structures could be preferable. When trained from scratch on the relatively small datasets considered here, the ViT underperformed relative to both the CNN and FNO on the evaluated tasks. Generally, transformer architectures have traditionally found success in settings where large datasets and extensive pre-training are available (Azad *et al.* 2023, Xu *et al.* 2024), both of which were absent in this study. The datasets examined here also represent a specific class of biological imaging problems characterized by relatively smooth, low-object-count population structures. While the architectural trends observed across these experimental and synthetic datasets provide evidence that inductive biases influence the spatial information learned by neural networks, future work could evaluate whether these conclusions extend to more structurally complex settings, such as sparse cell tracking and heterogeneous tissues. As our ability to image cellular dynamics expands to the scale of entire organisms (Daetwyler *et al.* 2025), this principle of matching the model to the scientific question becomes even more critical for meaningful discovery. Ultimately, our results provide evidence that researchers should deliberately select computational tools that match the spatial scale of their scientific question, moving beyond a one-architecture-fits-all approach to deep learning in biological image analysis.

### 4.2 Challenges in training biological representations

This study underscores an identifiability-adjacent problem when training deep learning models on biological images: a model’s predictive success does not guarantee a unique or mechanistically grounded interpretation of the underlying system. We find, e.g. that a CNN can correctly classify gastruloids by focusing on local cell clustering, whereas an FNO achieves nearly identical accuracy by learning their global morphology. Compounding this issue, we also observe that reconstruction fidelity, a common metric for representation quality in self-supervised learning, is a poor predictor of performance on subsequent biological tasks. This finding suggests that, while reconstruction loss remains a useful optimization objective, it may be insufficient as the sole criterion for selecting self-supervised models intended for downstream biological analysis. Together, these results indicate that model validation based on a single metric may be insufficient for reliable model selection, and that multiple criteria or tasks, where possible, would be beneficial. Alternative self-supervised approaches, such as contrastive objectives or masked prediction (Richter *et al.* 2024) may help encourage more robust or interpretable representations and could be the focus of future work. More broadly, addressing this validation challenge would benefit from open, FAIR (findable, accessible, interoperable, and reusable) (Wilkinson *et al.* 2016) standardized testing suites that evaluate learned representations more holistically with benchmark datasets that enable consistent and reproducible model comparisons. Efforts to establish such data resources are already underway (Horn *et al.* 2021, Nelson *et al.* 2021); benchmarks that capture the spatiotemporal complexity of modern imaging are still needed. This work contributes to these efforts by providing additional open data for testing models on population-level dynamics.

### 4.3 Inductive bias as a form of domain knowledge

The challenge of extracting meaningful multiscale information from increasingly high-resolution imaging datasets is a central theme in modern quantitative biology. In a field driven by the pursuit of mechanistic understanding, a model’s utility often depends not only on prediction accuracy but on whether its learned symbols, layers, and cost functions have biological interpretation. Our work highlights that a model’s inductive bias shapes the spatial scale of the learned features and the resulting interpretations. These findings are based on smooth, low-object-count imaging data, and the relative strengths of each architecture may differ for biological systems with other spatial organization.

One successful approach for incorporating domain knowledge is the use of physics-informed neural networks (PINN), which embed known governing equations into the model’s loss function. In biology, however, such first-principle equations are often unknown, intractable, or poorly defined. Therefore, an alternative strategy is to incorporate domain knowledge directly into a network’s architecture. Neural operators and other emerging network architectures—such as graph neural networks (Zhou *et al.* 2021, Li *et al.* 2023b) and geometric deep learning (Bronstein *et al.* 2021, Pineda *et al.* 2023)—provide a means to do so. As omics technologies improve, it is increasingly possible to incorporate transcriptomics and other molecular data into models as constraints or features; combined imaging and sequencing analysis has already shown success in uncovering biological mechanisms (Govind *et al.* 2022). Leveraging bias-informed or hybrid architectures may support more interpretable learning where governing equations are unknown.

These architectural choices also involve practical tradeoffs beyond representation quality. Although architectures such as FNOs and PINNs can reduce the need for brute-force parameter scaling by embedding structural priors, these benefits do not necessarily translate into faster computation. In our experiments, the FNO incurred longer training and inference times than comparably sized CNNs, likely because operations such as the FFT are not yet as highly optimized on modern hardware as the dense matrix operations that underpin more conventional neural networks. Consequently, model selection should balance representational suitability with practical considerations and computational cost.

## Supplementary Material

vbag238_Supplementary_Data

## Data Availability

This research supports open science; data and code are made publicly available where possible. The tumor simulation dataset is available on Zenodo at https://doi.org/10.5281/zenodo.19361370. The gastruloid images and preprocessing script are available on Zenodo at https://doi.org/10.5281/zenodo.19373112.

## Code availability

The autoencoder framework codebase is available on Zenodo at https://doi.org/10.5281/zenodo.19672838. The ARCADE ABM v2.4 source code (used to generate the synthetic tumor images) is available on Zenodo at https://doi.org/10.5281/zenodo.10622155. All other relevant information can be found on GitHub under their respective repositories at https://github.com/bagherilab.
