# Supplementary material for: Inductive bias influences the spatial scale of biological features learned from images
Source: Bioinform Adv. 2026 Aug 17;6(1):vbag238. doi: 10.1093/bioadv/vbag238 (PMC13549382; doi:10.1093/bioadv/vbag238)
Supplement: Supplementary file 1 [file vbag238_supplementary_data.pdf]

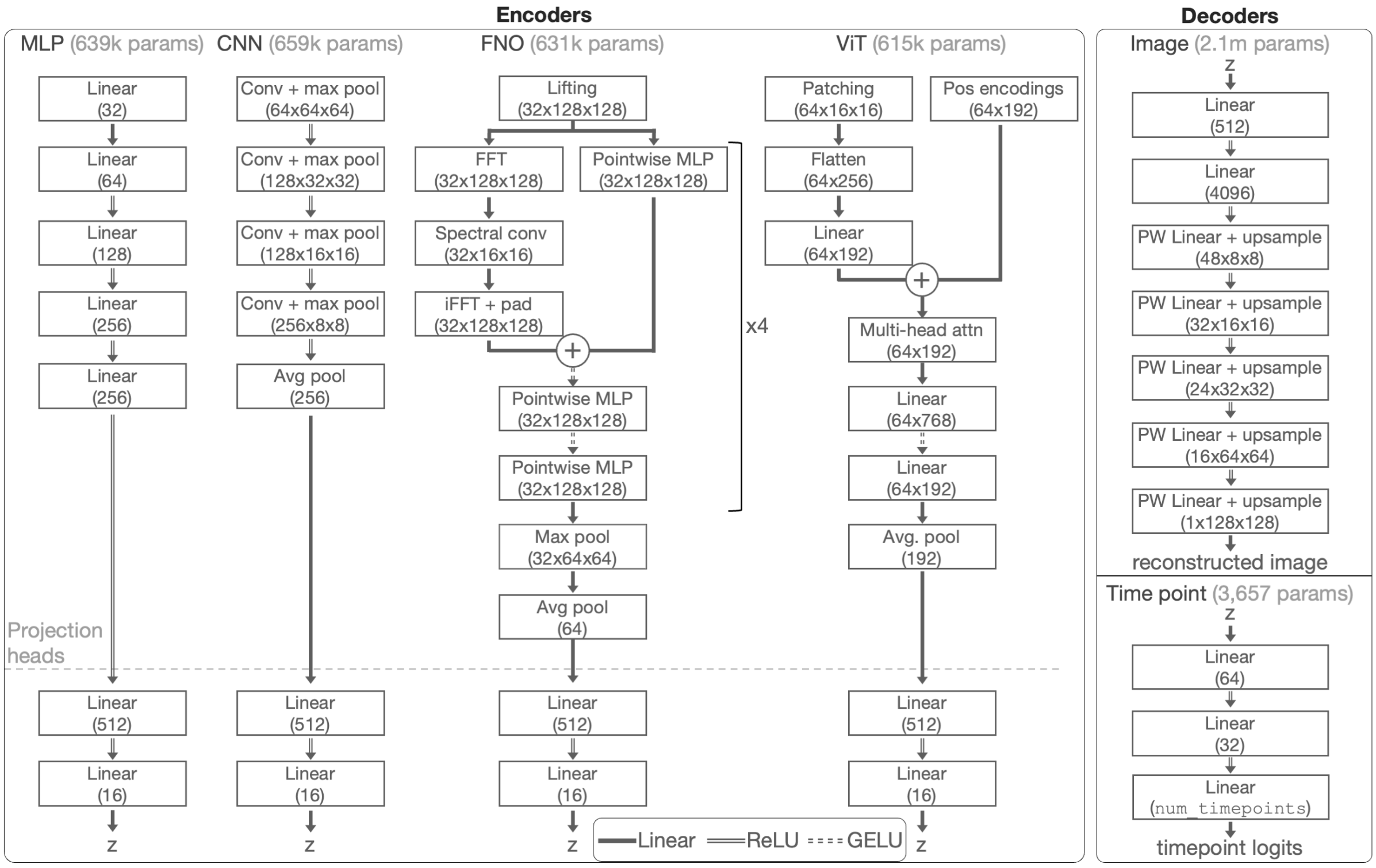

Supp. Fig. 1: **Comparison of autoencoder architectures**. Four different encoder architectures and trainable parameter counts are shown for each model type evaluated in this paper: MLP (639k params), CNN (659k params), FNO (631k params), and ViT (615k params). Each encoder is divided into distinct feature extraction layers and projection heads that are the same across models. The projection heads process the learned features down to the 16 dimensional latent space z. Both of the decoders are the same for all model variants and use the learned latent representations to predict either the original image in the case of the image decoder (2.1m params), or the relative time point the sample was from in the case of the time point decoder (3.657 params). Data output shapes for each layer (channels x height x width, or number of features) are indicated in parentheses under each layer name. For conciseness, all batch and layer normalization layers, as well as flattening and unflattening operations, have been excluded from the schematic.

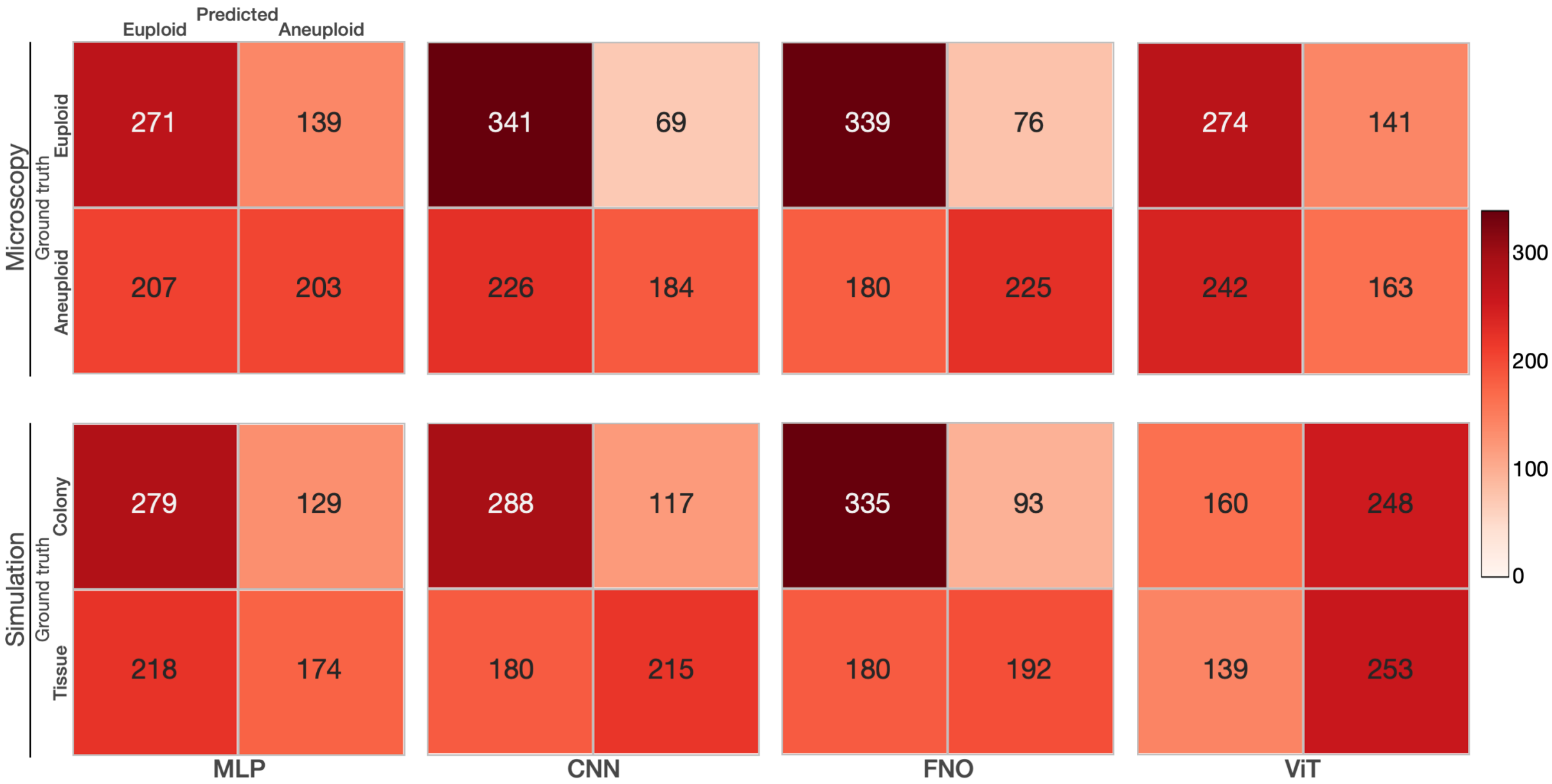

Supp. Fig. 2: **Trajectory classification confusion matrices.** Each 2×2 matrix shows the true-vs-predicted counts for trajectory classification for a model architecture aggregated over the training seeds ($n = 4$). Rows are true class and columns are the predicted class. Colors indicate counts over the pooled replicates.

**Dataset**	**Model**	**Learning Rate**	β_1	β_2
Microscopy	MLP	0.001	0.8	0.99
	CNN	0.001	0.8	0.99
	ViT	0.001	0.9	0.999
	FNO	0.001	0.8	0.99
Simulation	MLP	0.001	0.8	0.99
	CNN	0.0001	0.9	0.999
	ViT	0.0001	0.9	0.999
	FNO	0.001	0.9	0.999

Supp. Table 1: Selected hyperparameters for each model and dataset. Across all experiments, the Adam optimizer was selected, with the image loss weight (α) fixed at 0.9 and the latent dimension (d) set to 16.

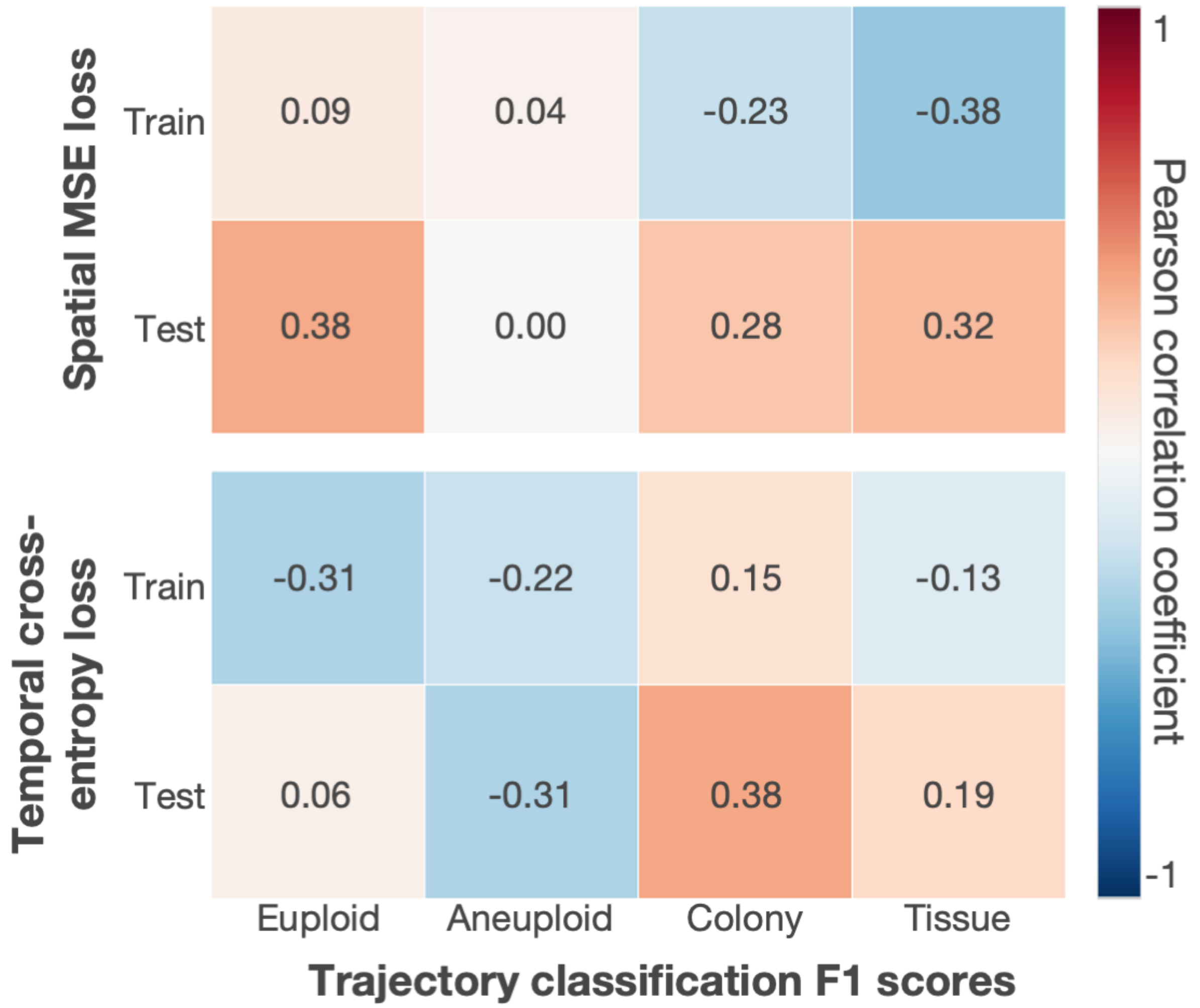

Supp. Fig. 3: **Correlation between reconstruction error and trajectory classification performance.** Heatmaps show Pearson correlation coefficients between reconstruction error and the per-class trajectory classification F1 score, computed across all architectures and random seeds ($n = 4$). Top: spatial image reconstruction MSE, bottom: temporal reconstruction cross-entropy error from encoder training. Color scale indicates Pearson correlation coefficient from -1 to 1.

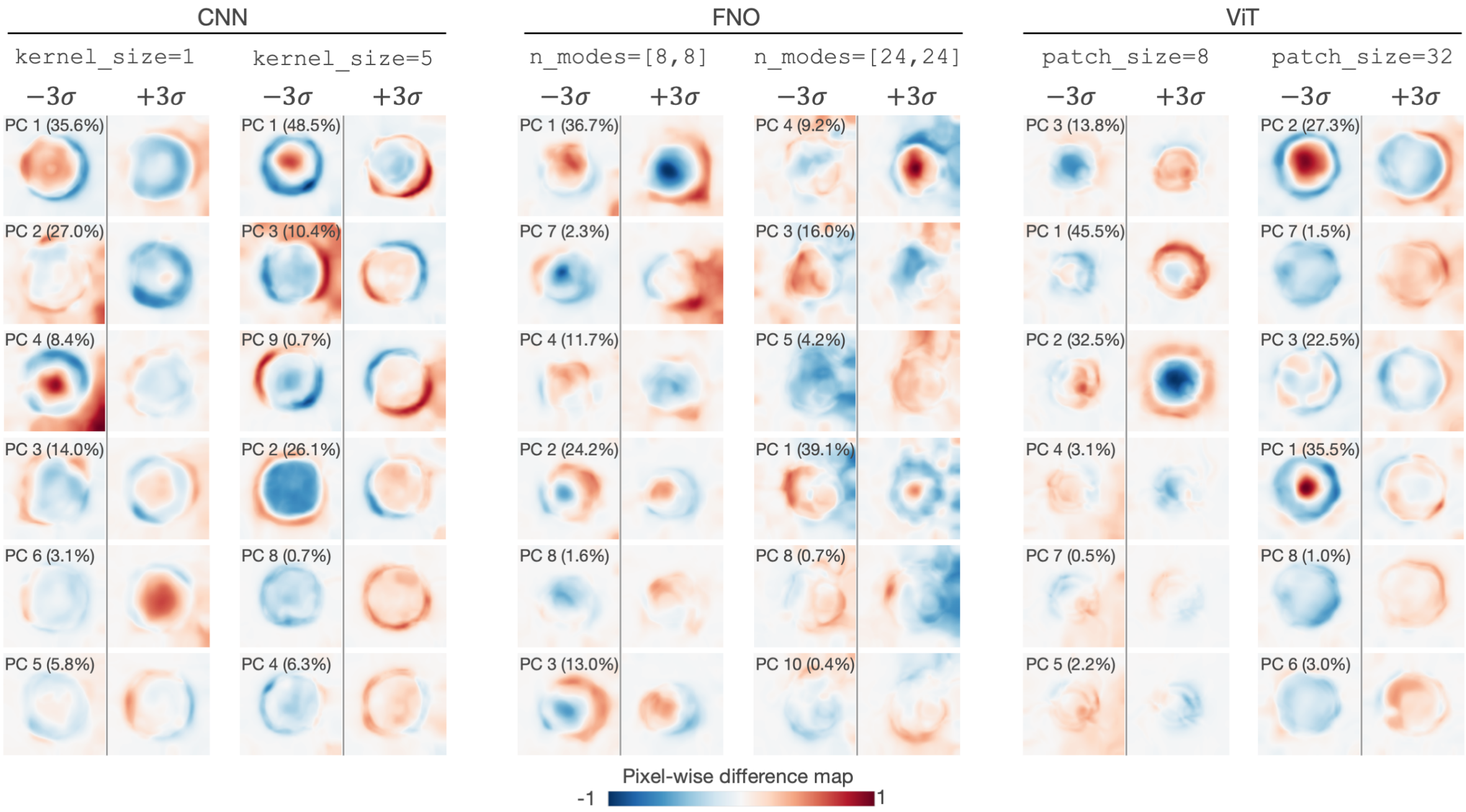

Supp. Fig. 4: **Hyperparameter robustness of PC counterfactuals.** PCs are ranked by predictive power for population classification using a permutation-based feature importance measure, and the six most predictive PCs are shown. Each PC is individually perturbed by ± 3 standard deviations (σ) and reconstructed through the image decoder. The result is visualized as a pixel-wise difference between the original and perturbed reconstructions (percent variance explained in parentheses). Each architecture is shown at with two hyperparameter settings lower and higher respectively than the ones used in the rest of the paper: CNN convolutional `kernel_size` (1 vs. 5), FNO Fourier mode count `n_modes` (8 vs. 24), and ViT `patch_size` (8 vs. 32). Note that direct comparison is difficult due to decreasing or increasing total model parameter count as a result of varying the hyperparameters.
